# Surface Modification of Metallic Inserts for Enhancing Adhesion at the Metal–Polymer Interface

**DOI:** 10.3390/polym13224015

**Published:** 2021-11-20

**Authors:** Libor Novák, Ladislav Fojtl, Markéta Kadlečková, Lukáš Maňas, Ilona Smolková, Lenka Musilová, Antonín Minařík, Aleš Mráček, Tomáš Sedláček, Petr Smolka

**Affiliations:** 1 Department of Physics and Materials Engineering, Faculty of Technology, Tomas Bata University in Zlín, Vavrečkova 275, 760 01 Zlín, Czech Republic; lnovak@utb.cz (L.N.); m1_kadleckova@utb.cz (M.K.); lmusilova@utb.cz (L.M.); minarik@utb.cz (A.M.); mracek@utb.cz (A.M.); 2 Centre of Polymer Systems, Tomas Bata University in Zlín, Třída Tomáše Bati 5678, 760 01 Zlín, Czech Republic; fojtl@utb.cz (L.F.); lmanas@utb.cz (L.M.); smolkova@utb.cz (I.S.); sedlacek@utb.cz (T.S.); 3 Department of Production Engineering, Faculty of Technology, Tomas Bata University in Zlín, Vavrečkova 275, 760 01 Zlín, Czech Republic

**Keywords:** insert, metal, polymer, adhesion, interface

## Abstract

A combination of mechanical and chemical treatments was utilized to modify the surface textures of copper and duralumin inserts in order to enhance the adhesion at the metal–polymer interface and provide an adhesive joint with a high loadbearing capacity. Pretreatment of the surfaces with sandblasting was followed by etching with various chemical mixtures. The resulting surface textures were evaluated with a scanning electron microscope (SEM) and an optical confocal microscope. Surface geometry parameters (Sa, Sz, and Sdr) were measured and their relationships to the adhesion joint strength were studied. It was found that the virgin and purely mechanically treated inserts resulted in joints with poor loadbearing capacity, while a hundredfold (duralumin) and ninetyfold (copper) increase in the force to break was observed for some combinations of mechanical and chemical treatments. It was determined that the critical factor is overcoming a certain surface roughness threshold with the mechanical pretreatment to maximize the potential of the mechanical/chemical approach for the particular combination of material and etchant.

## 1. Introduction

Components with a metal–polymer interface are widely used in many industries, namely the automotive and transportation industries. Such components consist of metal elements, known as inserts, which occupy a certain position in the component and are injection-molded with a polymer melt. Most of the component’s volume is occupied by a polymer [1,2]. Such hybrid components provide many advantages over standard parts, such as weight savings compared to purely metal parts, and superior mechanical properties compared to purely polymer parts. They can also outperform mechanically accomplished hybrid components, which must withstand inherent stress concentration from the holes for screws and rivets, as well as those aided by adhesives, which require additional curing time and/or specific curing conditions.

Despite the advantages of hybrid components with metallic inserts, there are several critical factors that must be taken into account: firstly, the precise placement of the insert in the mold and thus in the finished component; secondly, the issue of cold joints created by a slow flow of the melt into the mold and its gradual cooling. The precise placement of the insert is ensured by means of pins, supports, jaws, magnets, or a vacuum [1,2]. The formation of the cold joint can be avoided by adjusting the polymer flow, increasing the processing temperature, and adjusting the other process parameters. Another critical factor is adhesion at the metal–polymer interface. A strong adhesion is vital for transmittance of the forces acting on the resulting composite during its use.

In the case of metal–polymer joints, physical phenomena are responsible for the formation of the joint. These are hydrogen bonds and dispersion forces (London interactions). Hydroxyl groups are formed on the surface of the metal insert due to the reaction with water from the air, which results in the formation of hydrogen bonds between the metal surface and the polar plastic. Conversely, dispersion forces are responsible for the formation of the joint in the case of non-polar plastic.

One can highly enhance the quality of a metal–polymer joint by appropriate surface preparation of the metal insert, while keeping the processing parameters constant. The insert’s surface must be free of impurities, namely residual moisture or organic substances. The surface can be further oxidized, roughened, and activated during the pretreatment. Routinely used surface preparation methods include: cleaning and degreasing (using organic solvents and alkaline agents, degreasing with hot air or steam, ultrasonic cleaning, or plasma treatment), mechanical treatment (grinding, polishing, sandblasting, or shot peening), and chemical treatment (etching or soaking in acids) [3,4,5,6,7,8,9,10,11,12,13,14]. More elaborate techniques include coating, cataphoresis, electrical discharge machining, thermal arc spraying, laser structuring, or LAM (laser-assisted machining). Sadly, these techniques often require massive investment, which can render them prohibitive for many users. 

Recently, various approaches have been reported to solve the problem of achieving a reliable metal–polymer adhesive joint. Kajihara et al. applied abrasive jet blasting of the insert made of the aluminum alloy A5052 to obtain the most suitable surface microstructure. Glass beads and aluminum particles were used as abrasive materials. The highest shear strength was demonstrated by the samples with inserts blasted with aluminum particles [4]. Bonpain and Stommel investigated the effect of surface roughness on the shear strength of polymer (PA 66 + 30 GF) and aluminum (EN AW 3103) joints [6]. The samples were prepared in a shape used in the standard tests to assess the tensile strength of metals and polymers. It was demonstrated that if the surface roughness (Ra) was less than 10 microns, the adhesive failure of the sample occurred with no visible polymer residues on the aluminum surface. In contrast, when the Ra was higher than 10 microns, the cohesive failure was observed, i.e., the metal insert carried polymer residues. Gebhardt and Flesicher investigated the influence of an insert’s surface treatment on the tensile and flexural strength of the resulting component [5]. They applied two types of coating and five types of mechanical treatment, namely phosphate coating, cataphoretic painting, grit blasting, laser structuring, electroerosion, thermal arc spraying, and laser micro pins. The samples with a cataphoretically painted surface had a higher loadbearing capacity of the joint compared to the phosphate-coated surface and approximately the same loadbearing capacity as the samples treated with laser structuring. The highest loadbearing capacity was measured for samples with laser micro pins. Another way to modify the surface of the insert is sandblasting with corundum or silicon. Li, Gong et al. studied the effect of surface roughness, obtained by sandblasting, on surface wetting characteristics by examining contact angles [15]. They experimentally demonstrated that the wetting angle decreases as a result of decreased surface roughness, leading to better copying of the surface texture by the melt and, thus, to increased strength of the formed metal–polymer joint. Recent research shows that interest in enhancing adhesion at the metal–polymer interface is not limited exclusively to the automotive industry [16], and extends to the fields of biocomposites [17], impact-resistant materials [18], and heat exchangers [19]. Chemical modification of the polymer itself has also been reported [20].

The above-mentioned observations suggest that surface treatment of metallic inserts can significantly increase the loadbearing capacity of an adhesive joint; however, there is still a need for a rapid and straightforward method for surface modification/texturing that would not require prohibitively expensive tools and processes. The combination of mechanical and chemical approaches could be a pivotal step in achieving such a goal. The aim of this paper is to provide a series of guidelines for conducting this process. With respect to recent research data and our previous experience [10], a set of perspective etchants was chosen and applied to the surfaces of the metallic inserts. Purely physical (mechanical) methods were combined with chemical etching, where the etchant composition is vital for an effective etching process.

## 2. Materials and Methods

This section describes material selection and the process of testing specimen preparation, including the surface treatment of the metallic inserts. The testing procedures and evaluation techniques are also described here.

### 2.1. Materials

The materials used as inserts were duralumin (AW5754) and copper (CW004A). Standard reagents, including the chemical for etching, all in p.a. purity, were purchased from Sigma-Aldrich (St. Louis, MO, USA). Ultrapure water with resistivity 18.2 MΩ.cm was utilized (Direct-Q ^®^ 3UV, Merck, Darmstadt, Germany). The resin used for injection molding was TECHNYL^®^ A 218 V30 BLACK 21 NS (Solvay), which is a material extensively used in the automotive industry (PA 66 with 30% short glass fibers). 

### 2.2. Surface Treatment of the Inserts

Dog-bone shaped metallic inserts were laser cut from a 1 mm thick duralumin and copper sheets, degreased with acetone and left to dry, prior to further processing. The surfaces of the inserts were modified with both physical and chemical approaches as follows. The physical treatment involved sandblasting with either glass beads (Ballotini) or corundum (Al_2_O_3_) grit, designed SB-glass and SB-corundum, respectively, further in the text. Consequent treatment involved the application of various etching mixtures. These were Etch I (10 mL CH_3_OH + 10 mL HCl + 10 mL HNO_3_), Etch II (20 mL H_2_O + 9.8 mL HNO_3_ + 7.8 mL H_3_PO_4_ + 6 mL H_2_SO_4_ + 4 g NaNO_3_), Etch III (10 mL FeCl_3_ + 50 mL H_2_O), and Etch IV (10 mL HCl + 2 mL H_2_0_2_). The initial concentrations of the reagents were HCl 37%, HNO_3_ 65%, H_3_PO_4_ 85%, H_2_SO_4_ 96%, H_2_O_2_ 3%. The etching mixtures were prepared in a glass beaker and then conditioned at 25 °C for 12 h. The etching itself was performed at 25 °C for 120 s. Afterwards, the inserts were rinsed with an excess of deionized water, dried with compressed air, and stored in a desiccator.

### 2.3. Preparation of Testing Specimens

**Figure 1:**
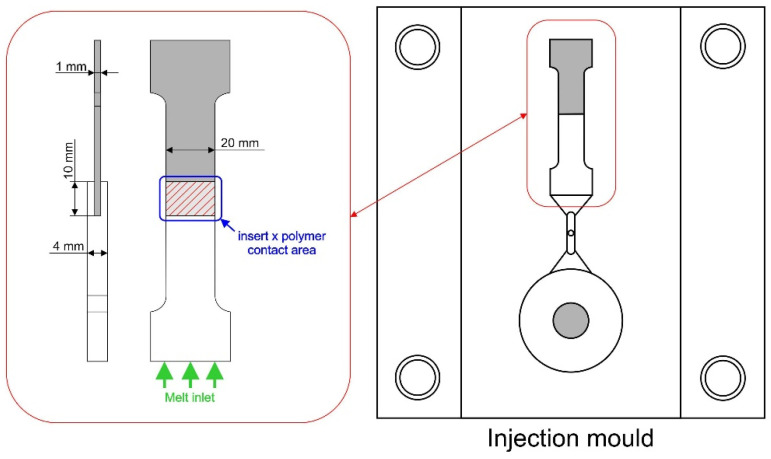
Dimensions of the testing specimen, contact area between the metal and polymer, melt inlet direction (**left**); detail of the mold cavity (**right**).

The specimens for testing the adhesion at the metal–polymer interface were prepared with the surface-treated metallic inserts and the Technyl resin in the 180MET III-15h (Mitsubishi, Nagoya, Japan) injection-molding machine with a double cavity mold. The process parameters were chosen in accordance with the resin manufacturer’s recommendations, i.e., temperature at individual barrel zones 260/270/260/250/240 °C, hot nozzle temperature 290 °C, mold temperature 60 °C, injection pressure 50 MPa, injection speed 50 mm/s, packing pressure 40 MPa, packing time 4 s, cooling time 20 s. Dimensions of the testing specimen and details of the mold cavity are displayed in Figure 1. 

### 2.4. Surface Roughness Analysis

Areal surface roughness parameters (Sa, Sz, Sdr) of metallic inserts were characterized with a VKX-1100 laser optical confocal microscope (KEYENCE CORPORATION, Mechelen, Belgium). Data analysis was performed with the Keyence MultiFileAnalyzer ver. 2.1.3.89 (KEYENCE CORPORATION, Mechelen, Belgium). Mean Sa (areal arithmetical mean height), Sz (areal maximum height–the sum of the largest peak height value and the largest pit depth value within the definition area) and Sdr (interfacial area ratio) values were determined from 5 individual measurements at various locations of a sample (each individual reading from the area of approximately 200 µm × 260 µm). The precise definition of the surface roughness parameters can be found elsewhere [21,22,23,24,25,26]. For the purpose of this paper, one can see Sa and Sz as the areal analogies of the Ra and Rz (arithmetical mean roughness and total profile height, respectively) profile parameters. The Sdr parameter, also called the area factor, falls among the so-called hybrid parameters. It represents the ratio between the interfacial and projected surface area (Equation (1)):

(1) $$S_{dr}=\frac{\left(Testured\;surface\;area\right)-\left(Cross\;sectional\;area\right)}{\left(Cross\;sectional\;area\right)}$$

### 2.5. Scanning Electron Microscopy

Surface topography of metallic inserts was characterized with a Phenom Pro (Phenom-World BV, Eindhoven, the Netherlands) scanning electron microscope (SEM). The samples were analyzed at the acceleration voltage of 10 kV in backscatter electron mode. 

### 2.6. Adhesion Testing

In order to evaluate the strength of adhesion at the metal–polymer interface, Testometric MT350-5CT universal testing machines (Testometric Company Ltd., Rochdale, UK), each equipped with a 5 kN load cell, were utilized. The tests were performed at room temperature (25 °C) at a crosshead rate of 10 mm/min. Five individual specimens were tested for each surface modification. For the sake of simplicity, adhesion strength is discussed as maximum force further in the text, as the contact area was identical for all samples. 

### 2.7. Statistical Evaluation

Where appropriate, respective standard deviations of the arithmetic mean for the 68.3% confidence interval are presented along with the arithmetic mean value.

## 3. Results and Discussion

Data from the SEM, confocal microscope, and universal testing machine are presented and discussed here to demonstrate the effect of physical and chemical treatment on the surface topography of the inserts and its effect on adhesion at the metal–polymer interface. 

### 3.1. Surface Treatment of the Inserts and the Resulting Surface Topography 

A combination of mechanical and chemical methods was chosen for the modification of the surfaces of the metallic inserts. Table 1 displays sample designation; Table 2 displays respective surface roughness parameters along with standard deviations.

**Figure 2:**
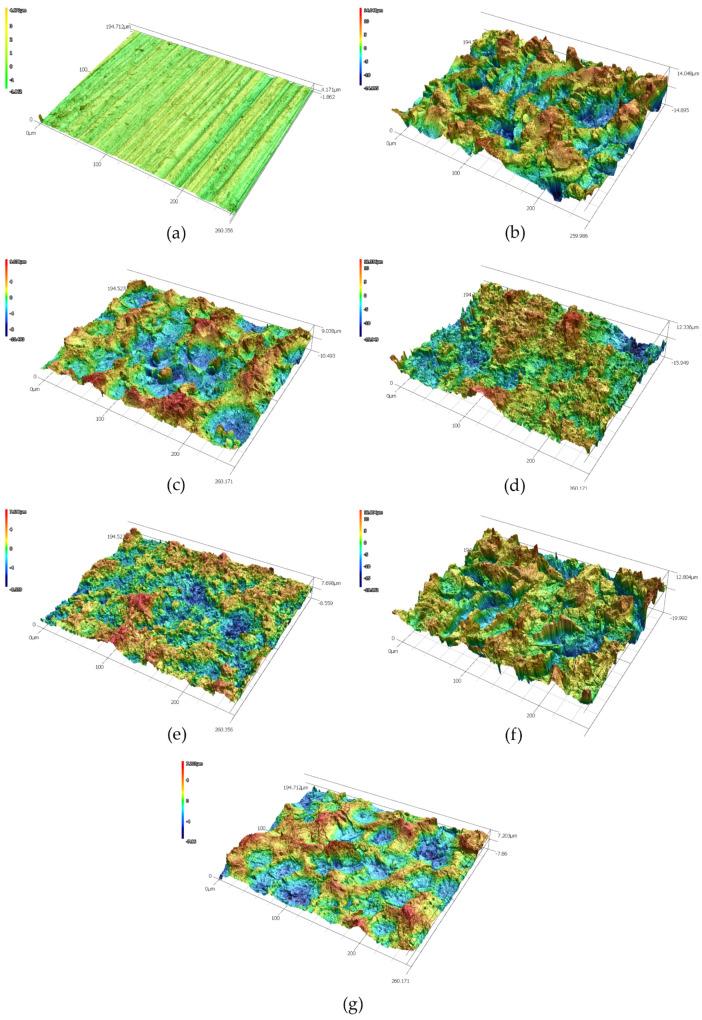
Duralumin samples and the effect of surface treatment on the surface structure. Laser confocal microscope micrographs: (**a**) virgin sample, (**b**) SB−corundum, (**c**) SB−glass, (**d**) SB−corundum + Etch I, (**e**) SB−glass + Etch I, (**f**) SB−corundum + Etch II, (**g**) SB−glass + Etch II.

**Figure 3:**
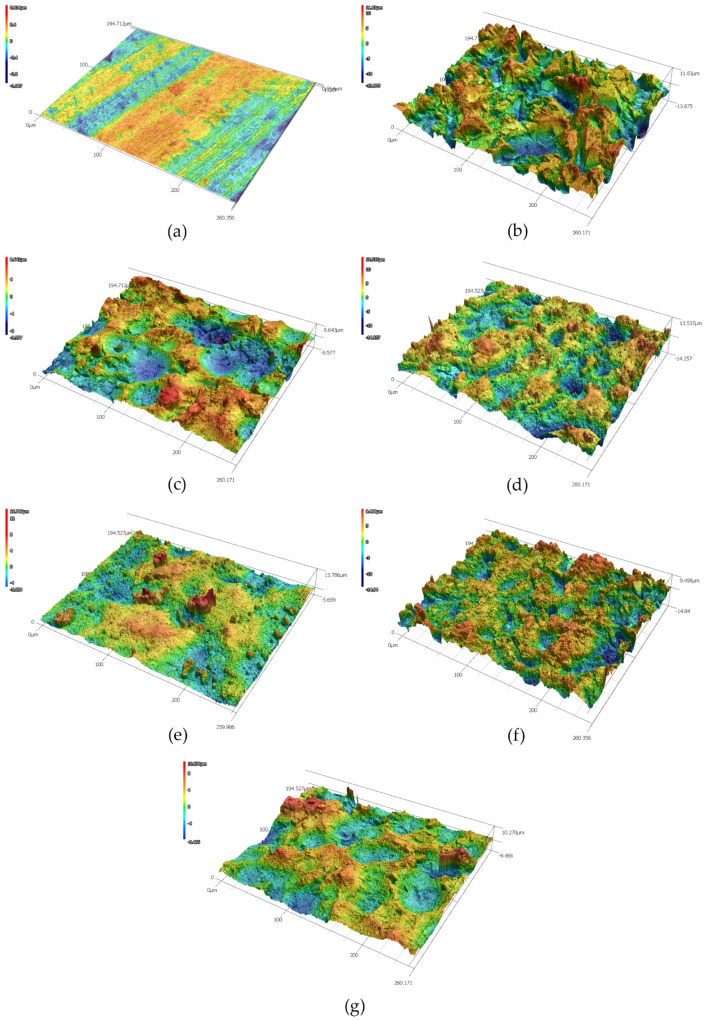
Copper samples and the effect of surface treatment on the surface structure. Laser confocal microscope micrographs: (**a**) virgin sample, (**b**) SB−corundum, (**c**) SB−glass, (**d**) SB−corundum + Etch III, (**e**) SB−glass + Etch III, (**f**) SB−corundum + Etch IV, (**g**) SB−glass + Etch IV.

The data in Table 2 show a severalfold increase in the Sa, Sz, and Sdr parameters in mechanically treated substrates, compared to original samples. One can also notice slightly lower roughness parameters in the copper substrates (Cu-1 and Cu-2) with respect to the duralumin substrates (Al-1 and Al-2). This could be attributed to the higher hardness of copper, but it is more likely due to the higher roughness of the virgin duralumin sample. The initial Sa values for duralumin and copper samples were (0.398 ± 0.013) µm and (0.154 ± 0.008) µm, respectively. As can also be observed in Figure 1, the duralumin virgin sample exhibits significant marks of the production process (rolling). As the standard areal parameters Sa and Sz (and by the same token, their profile counterparts Ra and Rz) very often do not reveal the whole truth about the surface texture, it is wise to take the Sdr parameter into consideration, particularly when adhesion enhancement is the primary target. Here, we see that the virgin samples exhibit only some 5% of the additional contact area compared to the idealized (flat) surface. From this point of view, one would expect the corundum-sandblasted samples, Al-1 and Cu-1, to be good candidates for adhesion enhancement, as their additional contact area has risen by approximately 180% and 115%, respectively, compared to the flat surface. Even better performance should be expected for the Al-5 and Cu-5 samples with approximately 230% and 140% increases, respectively. Figure 2 and Figure 3 show 3D images of the surface-treated duralumin and copper samples, respectively. These are in good agreement with the data presented in Table 2.

**Figure 4:**
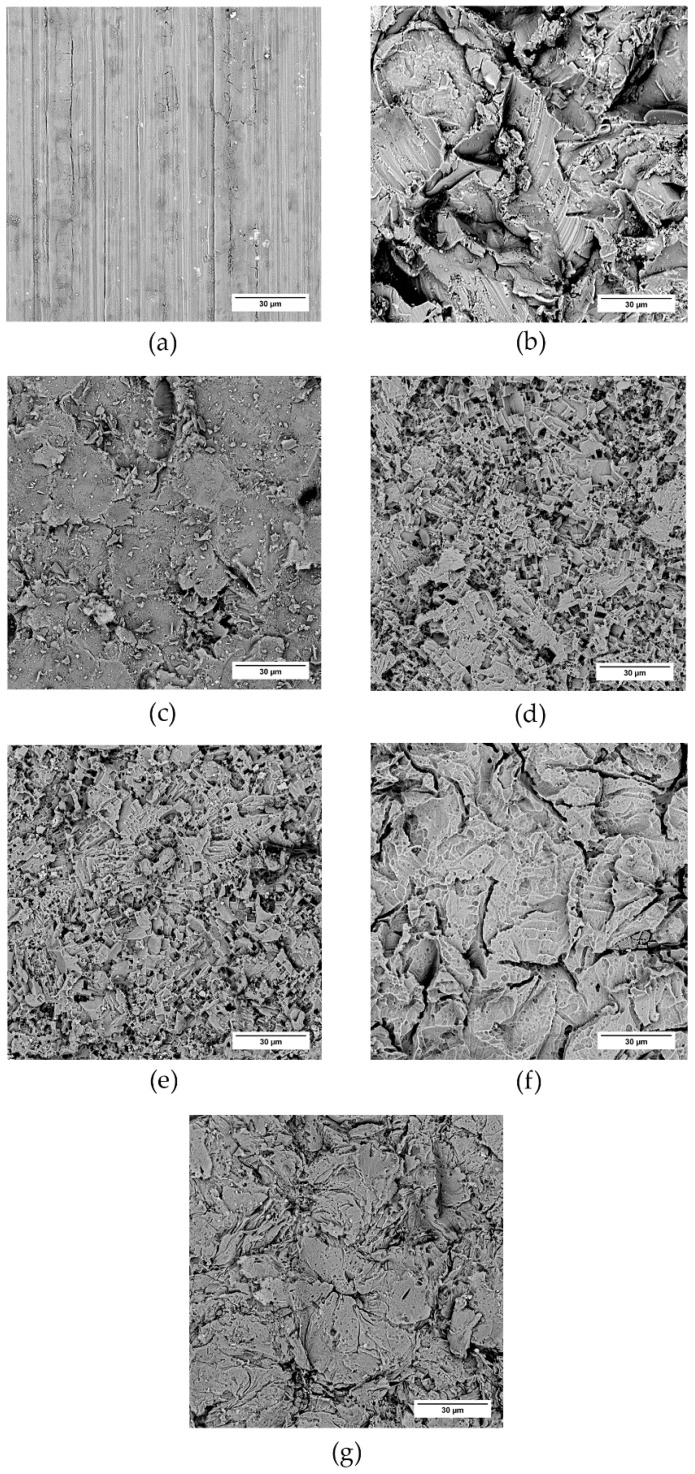
Duralumin samples and the effect of surface treatment on the surface texture. SEM micrographs: (**a**) virgin sample, (**b**) SB−corundum, (**c**) SB−glass, (**d**) SB−corundum + Etch I, (**e**) SB−glass + Etch I, (**f**) SB−corundum + Etch II, (**g**) SB−glass + Etch II.

**Figure 5:**
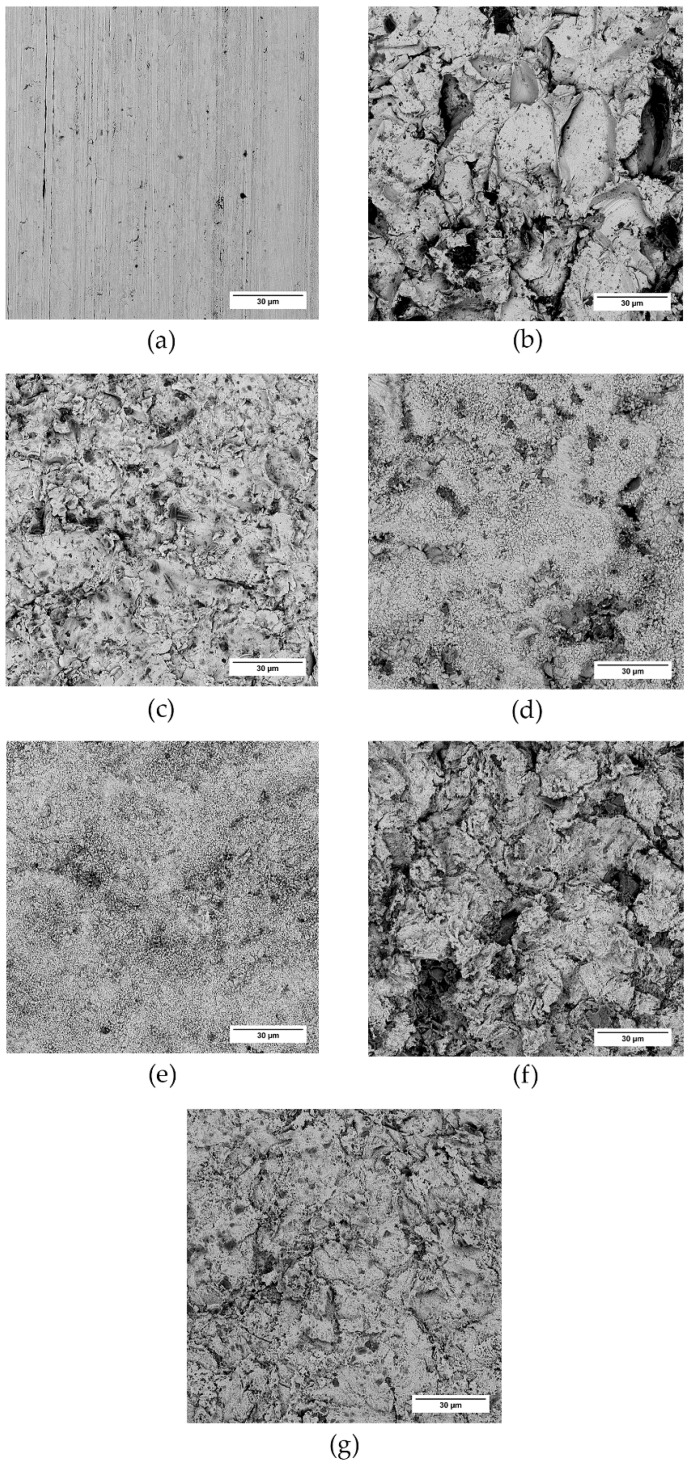
Copper samples and the effect of surface treatment on the surface texture. SEM micrographs: (**a**) virgin sample, (**b**) SB−corundum, (**c**) SB−glass, (**d**) SB−corundum + Etch III, (**e**) SB−glass + Etch III, (**f**) SB−corundum + Etch IV, (**g**) SB−glass + Etch IV.

The surface of the treated samples was further investigated with the help of SEM (Figure 4 and Figure 5). Again, both virgin samples bear signs of the production rolling process, and the corundum-sandblasted samples seem to exhibit the most profound changes in surface texture. This “pretreatment” also affects the surface texture after chemical treatment; we see that a much finer structure arises, superimposed onto the surface texture created earlier with the mechanical process. This way, a “composite” (hierarchical) structure can be created.

### 3.2. Adhesion

**Figure 6:**
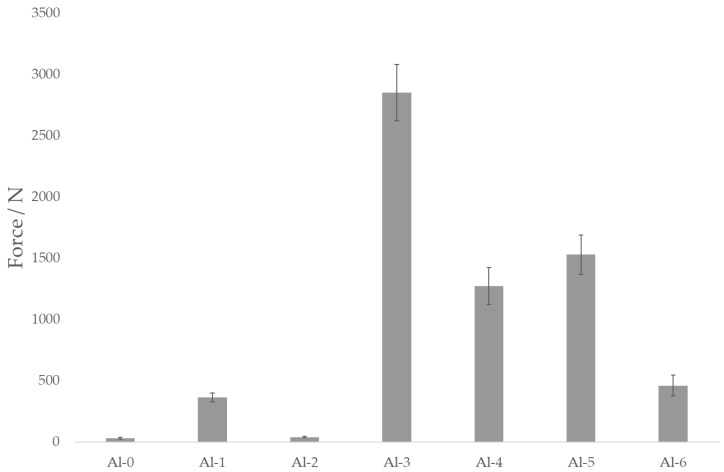
Duralumin samples and the effect of surface treatment on the adhesive joint strength. Samples designation according to Table 1.

**Figure 7:**
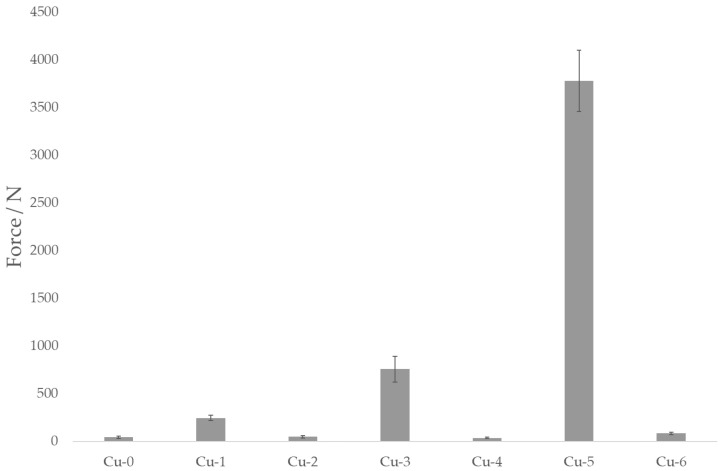
Copper samples and the effect of surface treatment on the adhesive joint strength. Samples designation according to Table 1.

The final adhesion between the metallic insert and polymer is one of the key factors in consistently forming an assembly with injection molding that can withstand the desired shear strain. Metals in general—and duralumin and copper in particular—suffer from the rapid formation of oxidized layers. When arising spontaneously, these can result in weak points that compromise the mechanical strength of the adhesion joint. Other sources of adhesion imperfections are contaminants, adsorbed gas or moisture, work-hardened layers, etc. [27]. The results of adhesion testing of the duralumin and copper inserts are shown in Figure 6 and Figure 7, respectively.

**Figure 8:**
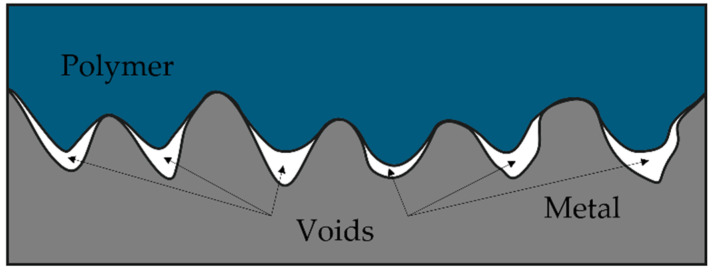
Rough metal–polymer interface. Improper setting of the injection molding process parameters.

As the data displayed in Figure 6 and Figure 7 show, adhesion between the polymer and non-treated metallic inserts is rather poor; the maximum forces are in the range of 30–40 N, meaning that such a joint could be disassembled simply by hand. Treating the inserts with corundum (samples Al-1 and Cu-1) results in a several-times increase in the adhesion force, though the samples treated with Ballotini glass beads (samples Al-2 and Cu-2) remain close to the values for the virgin samples. Here, one could rely on the roughness analysis as an indicator for predicting the adhesion strength; corundum-treated samples exhibit the highest values of all the roughness parameters (Table 2). Surface roughness parameter values are also higher in duralumin samples (Al-1 vs. Cu-1 and Al-2 vs. Cu-2), which is obviously related to the lower duralumin hardness compared to copper. The mechanical theory of adhesion would suggest that the higher the surface roughness (or the better the texture), the higher the adhesion strength (upon suppression of the formation of gas voids at the polymer–metal interface, with the proper setting of the injection molding process parameters [28], Figure 8).

Interestingly, other factors also play important roles in the formation of a strong adhesion joint. In the case of duralumin, treating the surface with corundum and subsequently with the Etch I (Al-3) results in an adhesion force of over 2800 N (almost an eightfold increase compared to the corundum-treated sample). The second highest adhesion strength is observed in sample Al-5 (corundum treatment followed with the Etch II), in which the force reached over 1500 N (a fourfold increase compared to the corundum-treated sample). As noted before, chemical etching can remove contamination present at the surface, loosely bound metallic particles or oxide layers, and superimpose a finer texture onto one formed with the previous step (sandblasting). The synergistic effect of higher surface roughness, superimposed fine texture, and chemical changes results in significantly higher adhesion strength compared to the virgin or purely mechanically treated samples. However, it seems that proper mechanical pretreatment is vital for achieving adhesion enhancement, as Ballotini-treated samples exhibit consistently lower adhesion strength when compared with corundum-treated counterparts (Al-3 vs. Al-4 and Al-5 vs. Al-6). The adhesion forces are approximately 2800 N vs. 1300 N and 1500 N vs. 500 N, respectively. We can observe similar pattern-etching with Etch I, which results in higher adhesion force compared to Etch II; notably, the surface texture changes to a rounder or more chamfered geometry with Etch II. However, it seems to be necessary to overcome a certain surface roughness threshold with the mechanical pretreatment to fully utilize the potential of the mechanical/chemical approach for this particular combination of material and etchants. In the case of copper inserts (Figure 8), the situation is somewhat similar. Pretreatment with Ballotini glass beads results in a surface roughness increase, but this change does not significantly reflect the adhesion strength. Except for the Cu-6 sample (ca. 80 N), the forces stay under the 50 N level. With corundum-treated samples (Cu-1, Cu-3, and Cu-5) we can see the adhesion force rise up to ca. 3800 N for the Cu-5 sample. As with the duralumin samples, proper mechanical pretreatment seems to be vital for achieving good adhesion with subsequent etching.

## 4. Conclusions

This work deals with the problem of adhesion at the metal–polymer interface. The aim of the research was to find a way of enhancing adhesion strength, and thus provide guidelines for the treatment of metallic insert surfaces with a combination of mechanical and chemical approaches. This topic is especially important in the automotive industry.

The investigation reveals that for a variety of reasons, the samples prepared with virgin duralumin and copper inserts exhibit very low adhesion strength, and such joints could be disassembled simply by hand (forces not exceeding 50 N in either case). Mechanical treatment with corundum and Ballotini (glass balls) increases the surface roughness and provides a cleaner surface for further modifications (once the surface has been cleaned of any sandblasting media), though only in the case of duralumin does it result in a significant rise of the joint adhesion strength. The best results were obtained from a combination of corundum mechanical treatment and proper chemical etching, where almost a hundredfold increase in the adhesion force value can be observed, compared to the virgin samples. A critical factor seems to be overcoming a certain surface roughness threshold with the mechanical pretreatment, to maximize the potential of a mechanical/chemical approach for the particular combination of material and etchant.

## Figures and Tables

**Table 1:** Sample designation.

Designation	Surface Treatment
Al-0	Virgin duralumin
Al-1	SB-corundum
Al-2	SB-glass
Al-3	SB-corundum + Etch I
Al-4	SB-glass + Etch I
Al-5	SB-corundum + Etch II
Al-6	SB-glass + Etch II
Cu-0	Virgin copper
Cu-1	SB-corundum
Cu-2	SB-glass
Cu-3	SB-corundum + Etch III
Cu-4	SB-glass + Etch III
Cu-5	SB-corundum + Etch IV
Cu-6	SB-glass + Etch IV

**Table 2:** Surface treatment, respective surface roughness parameters.

Designation	Sa (µm)	Sz (µm)	Sdr (-)
Al-0	0.398 ± 0.013	5.218 ± 0.302	0.051 ± 0.004
Al-1	3.139 ± 0.026	25.665 ± 1.003	1.833 ± 0.130
Al-2	2.386 ± 0.145	17.497 ± 1.055	0.694 ± 0.060
Al-3	2.278 ± 0.192	21.930 ± 1.834	1.392 ± 0.043
Al-4	1.665 ± 0.086	13.779 ± 1.099	0.718 ± 0.026
Al-5	3.411 ± 0.110	28.960 ± 1.641	2.310 ± 0.016
Al-6	1.738 ± 0.067	12.729 ± 0.676	0.383 ± 0.012
Cu-0	0.154 ± 0.008	1.499 ± 0.128	0.053 ± 0.003
Cu-1	3.007 ± 0.024	21.805 ± 1.140	1.155 ± 0.014
Cu-2	1.984 ± 0.217	14.367 ± 1.347	0.397 ± 0.009
Cu-3	2.144 ± 0.071	20.895 ± 2.336	0.748 ± 0.044
Cu-4	1.732 ± 0.057	15.648 ± 1.111	0.661 ± 0.017
Cu-5	2.369 ± 0.025	20.763 ± 1.115	1.414 ± 0.067
Cu-6	2.365 ± 0.269	16.130 ± 1.004	0.479 ± 0.051
